# Is Visual Creativity Embodied? Thinking Aloud While Performing the Creative Mental Synthesis Task

**DOI:** 10.3390/brainsci10070455

**Published:** 2020-07-16

**Authors:** Massimiliano Palmiero, Laura Piccardi

**Affiliations:** 1 Department of Human and Social Science, University of Bergamo, Piazzale S. Agostino, 2, 24129 Bergamo BG, Italy; 2 Department of Biotechnological and Applied Clinical Sciences, University of L’Aquila, 67100 L’Aquila, Italy; 3 Department of Psychology, Sapienza University of Rome, 00185 Roma, Italy; laura.piccardi@uniroma1.it; 4 Cognitive and Motor Rehabilitation and Neuroimaging Unit, IRCCS Fondazione Santa Lucia, 00185 Rome, Italy

**Keywords:** creativity, motor, embodiment, simulations, enacted, sensorimotor, body, visual, spatial

## Abstract

Over time, the view that creativity is embodied has emerged. In order to explore if visual creativity is supported by embodied mechanisms, the simulation approach was used as a framework of reference. The idea that visual creativity relies on mental representations that implement motor processes was faced. Participants were instructed to think aloud while carrying out the Creative Mental Synthesis Task, which allows to form pre-inventive structures and interpret them according to a specific category. Two independent judges scored verbal protocols in terms of the number of motor, spatial, and visual thoughts reported during the pre-inventive and inventive phases, and also evaluated the final objects according to originality and appropriateness. Originality was predicted positively by inventive motor thoughts and by pre-inventive spatial thoughts, but negatively by inventive spatial thoughts; appropriateness was only predicted by inventive visual thoughts. These results suggest that actions for future object utilization were simulated while interpreting pre-inventive structures, increasing originality of objects. In addition, spatial transformations are useful to construct the pre-inventive structures, but not to interpret them. Yet, thinking of the pictorial details of the object is also essential to classify it in a given category. Limitations and future research directions are discussed.

## 1. Introduction

Creativity is generally defined as the generation of original and appropriate ideas or products [1,2,3]. It can also involve surprise, which is a measure of how much new knowledge or expertise is acquired once the idea is created [4]. Creativity is also defined in terms of problem solving [5]. The implicit assumption of these definitions is that creativity is internal to the individual. However, creativity also relies on external factors, as it is developed in specific contexts and requires interactions with objects and materials [6,7]. This means that humans can create relying not only on mind processes but also on body and physical motion. 

The view that bodily movements shape the mind, and consequently creativity, can be interpreted within the frame of the embodied cognition theoretical approach [8,9,10]. This approach posits four basic principles: (1) the body, including brain and sensorimotor capabilities, supports cognition(embodiment) [11]; (2) organisms interact with both the physical environment on the basis of the possibilities (affordances) available [12,13] and the social-cultural environment (embedded) [14]; (3) cognition is for action, that is organisms form their experiences by initiating actions while interacting with their environment (enactive) [8,15,16,17]; (4) cognition is distributed beyond the body, that is non-biological devices (e.g., computers, smartphone, etc.) are used for many different functions that can strengthen mind and offer possibilities that differently could not be achieved (extended) [18]. Although the embodied approach makes a clear link between cognition, body, and action, the fully embodied accounts reject the concept of representation (e.g., the enactive and sensorimotor views), whereas the conservative embodied accounts retain it (e.g., the simulation view) for a review, see [19,20]. All accounts offer important implications for creativity.

On the one hand, the enactive account [8,15,16,17] focuses on the active ongoing exploration of the environment in order to seek for information which is important at a specific moment. Instead of generating a mental representation, the generation of a system of meanings by actions and interactions with the environment would be formed and used. Creativity would also be conceived as an adaptive action-based improvisational process, which occurs between creator and creation [9]. For example, the architect Olga Aleksakova claimed that she cut step by step a foam block to create a beautiful design for a building, without having in mind a clear representation of the final form [21]. Instead, the sensorimotor account [22,23,24] focuses on the mastery of knowledge of possibilities for action, defined as a set of rules of covariations between stimulation (input) and movement (output). Following this argumentation, creativity would rely on the ability to overcome usual sensorimotor laws, that is, on the capacity to build new knowledge about what new possibilities for action would be.

On the other hand, the simulation account [25,26,27] assumes that during experience association brain areas capture patterns of activation in sensory-motor areasin a bottom-up fashion, which are subsequently reactivated in a top-down fashion to implement perceptual symbols. Both storage and reactivation of perceptual symbols reflect single perceptual components rather than holistic perceptual experiences. Across experience, related perceptual symbols are integrated into a simulator, which is capable of producing limitless simulations, implementing all cognitive operations. This can go beyond experience in many ways. Therefore, creativity is operationalized as a mental representation that is used to simulate results, solutions, and alternatives [28], but also possible future actions in response to the creative output or object [29], producing new knowledge and opportunities. In line with this notion, both sensory and motor simulations would support creativity.

Different studies provided evidence that the motor system or body information is recruited while generating creative ideas not only in motor domain [30], but also in other domains, especially using the approach based on divergent thinking, which consists of thinking of already existing objects or ideas in many novel ways (e.g., alternative uses of common objects). At a behavioral level, walking was found to enhance verbal divergent thinking [31,32], probably activating metaphorical abstract concepts [31,33], stimulating executive functions, as well as the development of the reconfiguration of challenging problem spaces, and the formation of novel associations [34]. At a neural level, verbal divergent thinking tasks were found to yield activity specifically in the anterior intraparietal sulcus, implicated in organizing reaching and grasping [35], inferior parietal lobe, supramarginal gyrus, and precentral gyrus (the secondary motor area) (for a meta-analysis see [36]). This latter appears to be activated especially as a function of the rated creativity of ideas [37]. Visual divergent thinking was also found to correlate to the cortical thinness of supplementary motor areas and of higher areas of the prefrontal cortex [38].

Interestingly, the motor system appears to be involved also in creative product-oriented tasks. For example, musical improvisation was shown to be supported by motor and premotor cortices [39], such as the pre-central gyrus and the posterior cerebellum (for a meta-analysis see [40]), as well as by the posterior parietal lobe [41]. In addition, given the focus of the present study, higher involvements of both the supplementary motor area and dorsolateral prefrontal cortex were found while carrying out the visual creative synthesis task, consisting in mentally assembling three basic stimuli in order to create a recognizable object, as compared with a visuo-spatial control task [42]. Considering that responses were heterogeneous and not always involved the creation of an object at hand (e.g., human faces, animals, etc.), the activation of the motor system in such a study might not reflect necessarily action scenarios, but rather visuo-spatial transformation of stimuli. Although authors subtracted the visuo-spatial component, it should be noted that the creative task involved more complex spatial transformations than the visuo-spatial task, which still activated the motor system. In line with this interpretation, Sack et al. [43] found that during spatial imagery, involving the construction of an object, information (visual or verbal) upcoming from sensory cortex was sent to premotor cortex and then to parietal cortex, defining early and late functionally distinct stages of dynamic activations.

In light of this evidence, the extent to which visual creativity is embodied was explored. The framework of the simulation account was considered, based on the idea that visual creativity relies on mental representations that support an ongoing process that moves constantly backwards and forwards between generation of ideas, exploration, selection, and idea refinement [44] in accordance with internal abilities and external opportunities. This view is consistent with the Geneplore model of creative cognition [45,46], which underlines the key role of imagery [47,48,49], as an independent internal representation that supports the discovery of emergent structures and their possible functions. This model generally considers two basic creative phases, namely generative and explorative ones, that are repeated in a recursive fashion until the final idea is achieved. The generative phase involves the creation of a pre-inventive structure, by retrieving basic stimuli from memory and manipulating them. This phase is mostly based on spatial transformations. The explorative phase consists in interpreting the pre-inventive structure searching for implications, functions, and limitations. This phase is mostly based on conceptual inferences. This approach uses the general methods of cognitive science for the investigation of creativity, and also provides empirical support for many anecdotal accounts that have been reported by artists and scientists [50].

More specifically, using the logic underling the Geneplore model, this study explored if motor simulations (action-related thoughts) are involved in both generative and explorative phases of the visual creative process. The Think Aloud Method (TAM) was used, this method consists in verbalizing one’s thoughts aloud while carrying out a task. Although the TAM has limitations, such as the difficulty or impossibility to verbalize thought processes that operate unconsciously, the difficulty in verbalizing as quickly as thought processes occur, and the possibility that it increases the focus on irrelevant or incorrect ideas [51,52], it is considered a reliable source of information about thought processes [52]. Verbal reports produced by think-aloud data do not interfere with ongoing cognitive processing [53,54]. Comparing verbal divergent thinking (alternative uses task) carried out with TAM and without TAM, Gilhooly et al. [55] showed that fluency and novelty scores were similar across groups, confirming that no verbal overshadowing occurred while producing ideas. The TAM has been successfully used in different studies exploring product design [56,57] and drawing activity [58] involving creativity. Thus, the TAM can be considered a reliable approach to reveal numerous sub-processes that are actively involved in creativity.

The hypothesis was in line with the idea that motor simulations support the creative process not only during the construction of pre-inventive forms (generative phase), but also during the interpretation of them in order to achieve an actual invention (explorative phase). Given that the construction of pre-inventive forms does not require to think of a specific object, motor simulations were assumed to support the visuo-spatial transformations of stimuli; on the contrary, given that thinking of a specific object or instrument requires to using it in some way, motor simulations were assumed to be associated with new possibilities for action. Indeed, the explorative phase is situated, being determined by the ongoing interaction between the body and the object in a specific context.

## 2. Method

### 2.1. Participants

For this study 44 individuals from the Department of Biotechnological and Applied Clinical Sciences of University of L’Aquila were enrolled (mean age = 29.20, standard deviation = 4.53, age range = 21–38 years; 17 males and 27 females). From the anamnesis questionnaire, all participants were found to be healthy and without neurological and/or psychiatric disorders; no problem with alcohol or drug addiction was reported. In addition, participants were briefly interviewed and none of them declared to have a background in art or creative activities in general. Everyone signed the written informed consent after the procedures had been fully explained. The study was approved by the local ethical committee of University of Rome ‘Sapienza’ in May 2015 in accordance with the declaration of Helsinki.

### 2.2. Materials and Procedure

Participants took part in the study individually. They were firstly introduced in a quiet room where the informed consent was presented and the general procedure of the experiment was explained. Then, after signing the informed consent, participants were instructed to fill in the anamnesis questionnaire and were introduced to the Creative Mental Synthesis Task (CMST) [46] to be performed using the TAM. Participants were told that the aim of the task was to understand how people create objects using basic elements. For the TAM, the standard instructions adapted from Ericson & Simon [53,54] were used, see [55]. In order to get participants familiar with the CMST and the TAM, two practical trials were administered. In details, the CMST consisted of two distinct phases, each one lasting maximum 5 min. Firstly, during the pre-inventive phase, participants were instructed to form an abstract structure by mentally manipulating three components; each component could be rotated, made smaller, enlarged or stuck into one another, but not modified in the general structure. After the pre-inventive phase, a schematic drawing was produced. Secondly, during the inventive phase participants were asked to interpret the pre-inventive structure according to a pre-determined conceptual category (tools, sport goods, weapon) in order to create a definite object, considering also the materials, colors, and specific uses. Modifications of the general pre-inventive structure were not allowed, but it could be imagined bigger or smaller, or rotated with respect to the perspective drown on the paper sheet, and imagined in a spatial context while being used for a specific scope, for example, the screwdriver can be not only rotated but also embedded in something giving rise to a spatial interlocking. After the inventive phase, a brief explanation and a title for the object was provided. Basing on this procedure, participants were instructed to create three objects: one tool, one sport good, and one weapon. Finke’s [59] stimuli were used: sphere, half sphere, cube, cone, cylinder, rectangular block, wire, tube, bracket, flat square, hook, cross, wheels, ring, half ring. The three stimuli to be combined during the pre-inventive phase and the categories to be used during the inventive phase were chosen randomly across trials. During both phases, participants were asked to verbalize every thought aloud. They were asked to describe as accurately as possible inner thoughts involving the mental operations applied to stimuli, such as spatial transformation, rotation, embedding, moves in any direction, as well as to describe all visual mental images appearing in their mind’s eye, and any possible thought or image involving interactions with the stimuli and objects. Participants were also encouraged to report any other observation regarding emotions and reasoning processes. Participants were told to continuously think aloud. If they were silent for few seconds the examiner encouraged them to ‘keep thinking aloud’ while carrying out the task. Aside from this occasional reminder, there was no interaction between the examiner and the participants. The examiner recorded the verbal protocols for subsequent scoring. The experiment lasted approximately 45 min.

### 2.3. Scoring Procedures

Two independent and anonymous scorers (1 female, 25 years old; and 1 male, 35 years old) evaluated both verbal protocols and objects.

Regarding the verbal protocols, recordings were firstly transcribed and then evaluated one by one by the two scorers, separately, according to a coding scheme aimed at fitting with the nature of the CMST (visuo-spatial processing) and the embodiment issue under investigation (motor processing). Following Gu [60], first of all, the purpose was to reduce the large amount of qualitative data into meaningful and manageable patterns. Secondly, assuming the explanatory framework of the study, a top-down, theory-driven approach was used as a sort of guidance to what major themes to look for in the verbal protocols (e.g., motor, spatial, and visual processing). Scorers were instructed to evaluate the verbal protocols considering first the pre-inventive phase, then the inventive phase of the creative process. Using a marker pen, scorers had to identify information (mainly single words) in the transcripts that could be classified as thoughts that are motor, spatial, or visual in nature. In addition, although thoughts related to emotions and reasoning processing were not the main focus of the analysis, scorers were told to identify also this information in order to check for possible confounding factors that could affect the main stream of processing under investigation.

In order to correctly classify each type of thought, the following coding scheme was used:

(1) Motor thoughts had to include explicit motion-related verbs associated with stimuli manipulation and object use (e.g., move, shift, rotate, put, push, use, do) as well as all statements related to functionality of objects (e.g., it could be function as a screwdriver), based on the idea that motion-related information was implicit in such statements;
(2) Spatial thoughts had to include information containing spatial locatives (e.g., up/down, left/right, below/above, in/out, near/far, front/back) and spatial transformation (e.g., changes in size and rotation, embedding);
(3) Visual thoughts had to include information referred to basic visual features (e.g., rectangular block, sphere, cross), objects and parts of them imagined or created (e.g., water, glass, lamp, hat, car), colors (e.g., blue, red, white), materials (e.g., plastic, iron), and any other pictorial aspect (e.g., bright, dark);
(4) Emotional thoughts had to include any statement related to emotions (sadness, happiness, disgust, anger, surprise, fear) but also moods, such a s anxiety, discomfort, irritation, as well as appreciation, comfort, and pleasure;
(5) Reasoning thoughts had to include statements related to factual (e.g., if I do this then the object will be more sharp), counterfactual (e.g., if I had done like that, the object would have been better), or analytical (e.g., apply logic to find patterns or make inferences).

**Figure 1:**
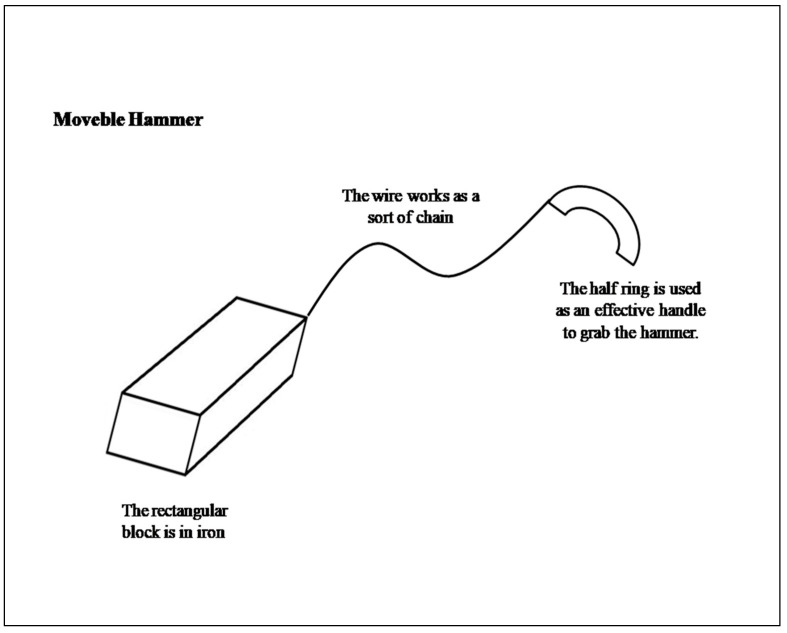
Note: Example of object created.

Some information was classified twice because belonging to two different types of thought, being part of different processes occurring simultaneously (e.g., ‘rotation’ involves both spatial thinking and also indirectly motor thinking). The approach to classify some information as two co-occurring types of thought was also used by Boldt [58], assuming that while creating some sub-processes occur simultaneously. As a practical example of the coding procedure (see Figure 1), on the one hand, given the stimuli ‘rectangular block’, ‘wire’, and ‘half ring’, the thought reported during the pre-inventive phase, such as ‘I am attaching the wire to the rectangular block, then the half ring, which is bigger is rotated rightward and attached to the extremity of the wire’, was coded as follows: for motor thought 2 points for ‘attaching/attach’ and 1point for ‘rotate’ (total 3 points); for spatial thought, 1 point for ‘rotate’, ‘rightward’, ‘bigger’, and ‘extremity’ (total 4 points); for visual thought, 2 points for ‘wire’, 1 point each for ‘rectangular block’ and ‘half ring’ (total 3 points). On the other hand, given the category ‘weapon’, the thought reported during the inventive phase such as ‘this object, defined as Movable Hammer, can be used to hits someone by the rectangular block in iron’; it is grabbed by the half ring and thrown, as a sort of ‘chainstick’ was coded as follows: for motor thoughts, 1 point each for ‘hits’, ‘grabbed’ and ‘thrown’ (total 3 points); for spatial thoughts, 0 points; for visual thoughts, 1 point each for ‘objects’ (assuming that it was visualized), ‘someone’, ‘iron’, ‘rectangular block’, ‘half ring’, and ‘chainstick’ (total 6 points).

Separately, the two judges assigned one point to each word or statement identified according to the type of thoughts considered. In short, they had to add a point each time a motor spatial or visual thought was reported. This led to get two frequencies of occurrence for each type of thought, one frequency for the pre-inventive phase (pre) and one for the inventive phase (post). Considering that the emotional and reasoning thoughts were identified only in a few participants’ verbal protocols, such data were not considered in subsequent statistical analyses. The inter-rater correlations (intra-class correlation coefficient—absolute agreement) for the remaining frequencies were significant for both the pre-inventive phase, in terms of ‘pre-motor’ (alpha = 0.80, *p* < 0.0001), ‘pre-spatial’ (alpha = 0.86, *p* < 0.0001), and ‘pre-visual’ (alpha = 0.81, *p* < 0.0001) thoughts, and the inventive phase, in terms of ‘post-motor’ (alpha = 0.75, *p* < 0.0001), ‘post-spatial’ (alpha = 0.93, *p* < 0.0001), and ‘post-visual’ (alpha = 0.88, *p* < 0.0001) thoughts. For each type of thought the average of the frequencies of occurrence defined by the two scorers was taken as the final score.

Regarding the final objects, following Amabile’s [61] consensual assessment technique, the scorers evaluated separately each of them in terms of originality, defined as an invention being new and not derived from something else, and appropriateness, defined as something being suitable and proper for a specific context. For each of the three objects, the scores varied from a minimum of 1 (very poor originality/appropriateness) to a maximum of 5 (very high originality/appropriateness). For example, the object was scored 1 in terms of originality when it was well known and did not include any new element or application (e.g., a screwdriver), 5 when it was unknown and involved a new application (e.g., a sport good capable to train and relax cervical muscles); the object was scored 1 in terms of appropriateness if it did not fit within the context proposed (e.g., a chisel with the cutting part was not sharp enough for being used to shape the stone or the wood), 5 when it really fit within the context (e.g., a tool with all parts harmonically assembled to be reasonably suitable, such as a funnel). The inter-rater correlations (intra-class correlation coefficient—absolute agreement) were significant for both ‘originality’ (alpha = 0.75, *p* < 0.0001) and ‘appropriateness’ (alpha= 0.78, *p* < 0.0001). For both parameters, the average of the evaluations defined by the two scorers was taken as the final score.

### 2.4. Statistical Analyses

Two hierarchical regression analyses were carried out, one for each dependent variable: originality and appropriateness. For both analyses two blocks of independent variables were used, that is, frequencies of pre-motor, pre-spatial, and pre-visual thoughts computed for the pre-inventive phase, and frequencies of post-motor, post-spatial, and post-visual thoughts computed for the inventive phase. Thought frequencies for the pre-inventive phase were entered first (first model), followed by thought frequencies for the inventive phase (second model).

## 3. Results

Descriptive statistics are presented in Table 1.

Firstly, possible multicollinearity among predictors was checked out. Based on the suggested cut-off value of 5 for the variance inflation factor (VIF) see [62,63], no multicollinearity was found, the VIF value ranging across predictors from 1.105 to 1.721, considering both models. Correlations between the predictors (two-tailed) are presented in Table 2.

Regarding the originality score, the first model was significant ((3,40) = 3.879 *p* = 0.016) and explained 22.5% of variance (*R*^2^ = 0.225, *R*^2^ Adj = 0.167). The predictors pre-spatial (β = 0.512, *t* = 2.946, *p* = 0.005) was significant, whereas the predictors pre-motor (β = −0.089, *t* = −0.556, *p* = 0.58) and pre-visual (β = −0.005, *t* = −0.033, *p* = 0.97) thoughts were not significant. The second model was also significant (F(6,37) = 3.776, *p* = 0.005) and explained 37.9% of variance (*R*^2^ = 0.38, *R*^2^ Adj = 0.279), that is an additional 15.4% of variance (*R*^2^ change = 0.154; F(37,3) = 3.071, *p* = 0.040). In the second model the predictors pre-spatial (β =0.538, *t* = 3.165, *p* = 0.003), post-motor (β = 0.325, *t* = 2.386, *p* = 0.022) and post-spatial (β = −0.332, *t* = −2.234, *p* = 0.032) thoughts were significant, whereas the predictors pre-motor (β = −0.069, *t* = −0.461, *p* = 0.65), pre-visual (β = −0.022, *t* = −0.131, *p* = 0.90) and post-visual (β = 0.043, *t* = 0.271, *p* = 0.79) thoughts were not significant.

Regarding the appropriateness score, the first model was not significant (F(3,40) = 1.102, *p* = 0.36). The second model was significant (F(6,37) = 2.699, *p* = 0.028) and explained 30.4% of variance (*R*^2^ = 0.304, *R*^2^ Adj = 0.192), that is an additional 22.8% of variance (*R*^2^ change = 0.228; F(3,37) = 4.044, *p* = 0.014). In the second model, only the predictor post-visual thoughts (β = 0.361, *t* = 2.135, *p* = 0.039) was significant, whereas the predictors pre-motor (β = −0.311, *t* = −1.953, *p* = 0.06), pre-spatial (β = −0.139, *t* = −0.774, *p* = 0.44), pre-visual (β = −0.166, *t* = −0.935, *p* = 0.36), post-motor (β = −0.064, *t* = −0.446, *p* = 0.66), and post-spatial (β = −0.30, *t* = −1.911, *p* = 0.06) thoughts were not significant.

## 4. Discussion

In the present study, the extent to which visual creativity is embodied was explored in light of the simulation account, assuming that creativity relies on mental representation-based simulations that implement not only spatial and visual processing but also motor processing. To this purpose, participants were instructed to think aloud, that is to externalize their thoughts verbally while creating objects by the Creative Mental Synthesis Task (CMST), which allows firstly to form pre-inventive structures and then to interpret them according to a specific category. Verbal production allowed to determine motor, spatial, and visual thoughts during both the pre-inventive and the inventive phases. Final objects were evaluated in terms of originality and appropriateness. Results showed that originality was predicted positively by spatial thoughts reported during the pre-inventive phase. During the inventive phase, originality was instead predicted negatively by spatial thoughts and positively by motor thoughts, whereas appropriateness was only predicted by visual thoughts reported during the inventive phase.

These results highlighted that visual creativity can be embodied to some extent because motor processing was found to support originality of objects. Given the nature of the task, it is highly plausible that actions simulated while interpreting the pre-inventive structures played a functional role in mentally generating possible future uses of the objects created [29]. This means that the generation of motor simulations in this study does not reflect the visuo-spatial transformation of stimuli for two reasons: firstly, stimuli were transformed in the first phase of the creative process, during the formation of the pre-inventive structures; secondly, the categories to which the pre-inventive structures had to belong to referred to objects at hand, such as tools, weapons, and sport goods, which generally involve action scenarios to be used. In other words, motor thoughts took part in the goal-directed planning of objects, or interpretation of pre-inventive structures, by simulating actions, which in turn yielded an enhancement of originality. Considering the initial hypothesis, this result confirms that motor simulations played a key role in the exploration phase, but not in the generative phase of the creative process, supporting the view that embodied mechanisms can also support conceptual interpretation, contextual shifting, structure searching for functions and limitations. Nevertheless, it should be clarified that motor thoughts were not found to predict the appropriateness of objects. Given that this attribute was evaluated as something suitable and proper for a specific context, with clear implications for visual processing, it might be that judges did not consider information for action possibilities when evaluated it. Interestingly, appropriateness was predicted by visual thoughts, as if the suitability of creative objects to be part of a specific category would depend on both the visualization of the object itself and pictorial information. Thus, it would be more important to mentally explore the details of the object rather than to think of possible actions that could be performed in order to place the object within a specific category.

A case apart is the issue of the spatial thoughts. Originality was predicted positively by spatial thoughts reported in the pre-inventive phase, but negatively by spatial thoughts reported in the inventive phase. On the one hand, this result shows that spatial transformations applied at the beginning of the creative process are essential to get high originality in the subsequent phase. On the other hand, spatial transformations applied to the whole object seem to dampen originality. Although the positive result is plausible, the negative one conflicts with Palmiero et al.’s [64] study, which found that the transformation ability predicted the originality of inventions. Nevertheless, it should be noted that in the present study the transformation ability was not assessed, participants only reported the occurrence of their spatial thoughts. It might be that when participants reported to apply spatial transformations to the final object, originality was negatively affected because the interpretation changed or adapted to a more conventional idea (at this stage, participants could not change the general pre-inventive structure, but could imagine it bigger or smaller, view it from different perspectives, and imagine the object in a spatial context while being used for a specific scope).In this direction, participants might have reported only simple spatial transformations. Whether or not implicit spatial transformations more compatible with originality were actively at work during the interpretation of the final object is unclear. Undoubtedly, this issue deserves more study.

In general, this study showed the link between body and visual creativity. Simply thinking of or simulating physical object-manipulations may liberate processes essential to originality. This is in line with those studies that found an association between the motor system and divergent thinking, which is basically based on finding alternative uses of common objects [35,36]. Following Benedek et al. [34], embodied behaviors might facilitate the stimulation of executive functions, for example inhibition, and mental set-shifting, help the development of abstract thought schemas and the formation of novel associations. This would explain why physical object manipulations enhance divergent thinking even though the object manipulated is different from the stimuli used to assess divergent thinking [65]. In this vein, it should be outlined that motor simulations are supported by mental imagery, regardless of the nature of the task. Even when solving alternative use tasks which do not require explicitly to use imagery, people spontaneously create mental images that represent situations or objects, which provide people with action simulations. Mental representations of the environment are crucial for the control of action because they define spatial outlines of action possibilities [66]. In particular, visual imagery contributes to action representation in two ways: when imaging someone else performing an action, that is in the visual motor imagery involving the third-person perspective, and when imaging ourselves performing an action, that is in visual motor imagery involving the first-person perspective [67]. In the present experiment, even though no specific instruction was given, it is highly probable that participants used the internal perspective while complying with the task requirements. Future studies should clarify this issue, as well as the extent to which motor thoughts reflect the use of the kinesthetic motor imagery modality, based on the feeling of the movement from a first-person perspective [67].

Despite this study shedding light to some extent on the embodied mechanisms that support visual creativity, the result is limited to objects at hand and appears to be task dependent. In some kinds of creative tasks, such as in the CMST as used here, targets and emerging means co-evolve [56]. Therefore, in future studies, it might be interesting to assess the role of body and action while creating less practical objects (e.g., pixies, flying saucers, etc.). Moreover, beyond originality and appropriateness one might consider also different dimensions or characteristics of creativity that could also implement embodied mechanisms, such as aesthetic qualities of products. Yet, as clarified above, although the TAM can be a useful tool to assess inner thought process, it obviously cannot extrapolate the whole reasoning and strategies used to carry out the task, especially in non-trained individuals, and it does not indicate anything about the level of individuals’ ability (one can declare to be using a specific process but it does not mean that the underlying ability is possessed). Therefore, in subsequent studies it could be useful to combine the subjective TAM with more objective methods. Finally, the simulation account has been used as a theoretical framework of reference. However, one could also investigate in which way creativity relies on active ongoing exploration of the environment (enactive approach) or on the mastery of knowledge of possibilities for action (sensorimotor approach), without being supported by mental representations. To our best knowledge, only verbal anecdotes are available, such as Olga Aleksakova’s enactive one [21], above mentioned. The situated perspective should be also considered, in order to understand how creativity gains advantage by interacting with the physical environment on the basis of affordances available, and how it is embedded in socio-material environments [9]. This would allow us to study creativity as a process dynamically supported by person–environment (both physical and social) interactions.

In conclusion, despite the evidence for embodied mechanism to keenly enhance visual originality of objects at hand, much research is needed to clarify the link between body and creativity, with relevant implications also for pedagogical and rehabilitation practices with both normal and pathological samples.

## Figures and Tables

**Table 1:** Descriptive statistics.

	Mean	S.D.	Min	Max
Originality	1.3	1.1	0	4.7
Appropriateness	3.3	0.90	0.50	5
Pre-motor	0.53	0.29	0.08	1.6
Pre-spatial	0.81	0.56	0.06	2.4
Pre-visual	3.3	1.2	1.1	7.6
Post-motor	0.54	0.34	0.08	1.3
Post-spatial	1.4	0.76	0	4
Post-visual	3.8	1.1	1	6

Note: Pre- = Pre-inventive scores; Post- = Inventive scores. S.D. = Standard deviation.

**Table 2:** Correlations among predictors.

	Pre-Motor	Pre-Spatial	Pre-Visual	Post-Motor	Post-Spatial	Post-Visual
Pre-motor	1					
Pre-spatial	0.467 **	1				
Pre-visual	0.370 *	0.522 **	1			
Post-motor	0.094	0.152	−0.054	1		
Post-spatial	0.212	0.281	0.016	0.257	1	
Post-visual	0.340 *	0.416 **	0.434 **	0.151	0.368 *	1

Note: significance two-tailed; ** *p* < 0.01; * *p* < 0.05; Pre-inventive; Post- = Inventive.
